# First-pass perfusion CMR two days after infarction predicts severity of functional impairment six weeks later in the rat heart

**DOI:** 10.1186/1532-429X-13-38

**Published:** 2011-08-03

**Authors:** Daniel J Stuckey, Carolyn A Carr, Stephanie J Meader, Damian J Tyler, Mark A Cole, Kieran Clarke

**Affiliations:** 1 Department of Physiology, Anatomy and Genetics, University of Oxford, Parks Road, Oxford, UK; 2 Biological Imaging Centre, Imperial College, Hammersmith Hospital, Du Cane Road, London W12 0NN, UK

## Abstract

**Background:**

In humans, dynamic contrast CMR of the first pass of a bolus infusion of Gadolinium-based contrast agent has become a standard technique to identify under-perfused regions of the heart and can accurately demonstrate the severity of myocardial infarction. Despite the clinical importance of this method, it has rarely been applied in small animal models of cardiac disease. In order to identify perfusion delays in the infarcted rat heart, here we present a method in which a T_1 _weighted MR image has been acquired during each cardiac cycle.

**Methods and results:**

In isolated perfused rat hearts, contrast agent infusion gave uniform signal enhancement throughout the myocardium. Occlusion of the left anterior descending coronary artery significantly reduced the rate of signal enhancement in anterior regions of the heart, demonstrating that the first-pass method was sensitive to perfusion deficits. *In vivo *measurements of myocardial morphology, function, perfusion and viability were made at 2 and 8 days after infarction. Morphology and function were further assessed using cine-MRI at 42 days. The perfusion delay was larger in rat hearts that went on to develop greater functional impairment, demonstrating that first-pass CMR can be used as an early indicator of infarct severity. First-pass CMR at 2 and 8 days following infarction better predicted outcome than cardiac ejection fraction, end diastolic volume or end systolic volume.

**Conclusion:**

First-pass CMR provides a predictive measure of the severity of myocardial impairment caused by infarction in a rodent model of heart failure.

## Background

Non-invasive, *in vivo *measurements of cardiac morphology, viability and function have revolutionised our understanding of cardiac disease [1-3]. In combination, echocardiography, angiography, PET, SPECT, CT and CMR can be used to measure wall and valve motion, cavity volumes, aortic and coronary blood flows, tissue perfusion and cardiac metabolism [1-5]. CMR offers excellent soft tissue contrast, coupled with high temporal and spatial resolution, allowing accurate measurement of cavity volumes, wall thickness and ejection fraction. Further, infarct size and transmurality, measured using CMR shortly after systemic infusion of the contrast agent gadolinium-diethylenetriaminepentaacetate (DTPA), strongly correlates with progression to heart failure and patient mortality [2,5-8]. MR imaging of the first-pass of a bolus infusion of Gd-DTPA has become a standard clinical method for identifying under-perfused regions of the human heart and accurately predicts the severity of myocardial ischemia [9-14].

Scaling down imaging techniques to study the ever increasing number of small animal disease models has been a formidable challenge [15]. The rat model of myocardial infarction, induced by occlusion of the left anterior descending coronary artery, is widely used and has provided data essential for the development of surgical and pharmacological therapy [16]. However, the left ventricular mass of the rat heart (~500 mg) is approximately three hundred times smaller than the human left ventricle (~150 g), and contracts 5 to 10 times faster, making it necessary to use high field strengths, smaller voxel sizes and rapid gating strategies.

Small animal cine-MRI has identified increased left ventricular mass, end diastolic and end systolic volumes in the infarcted heart during progressive cardiac remodeling [17]. Pharmocologiical and biological therapies have been extensively tested in this model system [16,18]. However, owing to the variability of infarct size induced by experimental myocardial infarction, it is useful to have a baseline measure of infarcted heart function prior to administration of the therapeutic agent. Acute changes to left ventricular mass and volume, typically made using echocardiography, are not good predictors of functional outcome, making these frequently used experimental methods for assessing acute infarct severity inaccurate. A more robust early measure of disease severity may be obtained from late gadolinium enhancement (LGE) CMR [19] or perfusion imaging. LGE-CMR identifies tissue damage due to increased contrast agent retention within infarcted areas of caused by cell membrane rupture and increased interstitial space [3,5]. These processes occur within hours of infarction, so offer good early predictors of infarct severity. An even earlier indicator of the extent of functional impairment may be offered by perfusion imaging because it is the reduced perfusion that ultimately results in cell necrosis, membrane rupture, reduced contractility, remodeling and heart failure [10]. Myocardial perfusion has been imaged using arterial spin labeling and T_1 _mapping [20-23], but imaging the first-pass of a Gd-bolus infusion has rarely been used to study small animal models of myocardial infarction [24,25], probably owing to the need for ultra-fast and high-resolution imaging [20].

Here, we have developed a method to identify perfusion deficits in infarcted rat hearts during bolus infusion by acquiring a T_1 _weighted MR image every cardiac cycle, both in the isolated perfused heart and *in vivo*. Perfusion delay and myocardial viability, morphology and function were measured *in vivo *at 2 and 8 days after infarction to compare with cardiac function measured in the same animals at 42 days.

## Methods

### Experimental design

For ex vivo MRI studies of isolated, perfused hearts, control female Wistar rat hearts (n = 12) were isolated, perfused and imaged as described below. Hearts were then removed from the magnet, the coronary artery occluded, the heart repositioned and re-imaged (n = 8).

For *in vivo *MRI studies, baseline cine-MRI was performed prior to surgery. Female Wistar rats (n = 34) underwent myocardial infarction followed by *in vivo *first-pass imaging, cine- and LGE-CMR at 2 and 8 days. Problems with i.v. infusions, owing to unsuccessful tail vein cannulation, movement of cannulae during positioning of rats within the magnet and blood clots or air bubbles in the drug line, meant that first-pass imaging could not be performed on all 34 rats at each time point (2 days, n = 21; 8 days, n = 27; both 2 and 8 days, n = 14). After 9 days, 7 of the rats that were imaged at 2 and 8 days were sacrificed; hearts were isolated, perfused and imaged *ex vivo*. The remaining 27 rats (14 and 20 of which had had first-pass imaging at 2 and 8 days respectively) underwent cine-MRI at 42 days. The University of Oxford Animal Ethics Review Committees and the Home Office (London, UK) approved all procedures.

### *Ex vivo *MRI of isolated, perfused hearts

Hearts were excised from control (n = 12) or infarcted (n = 7) rats, arrested in ice-cold Krebs-Henseleit buffer and attached to a cannula by the ascending aorta. Hearts were perfused at 85 mmHg with filtered Krebs-Henseleit buffer containing 11 mM glucose and 1.8 mM pyruvate. Buffer was maintained at 37°C and pH 7.4, aerated with 95% O_2_/5% CO_2 _and not re-circulated, to avoid contrast agent accumulation. A balloon connected to a pressure transducer was placed in the left ventricular (LV) cavity, inflated to an end diastolic pressure of 4 mmHg and used to measure cardiac function. The heart was placed in a 20 mm NMR tube and lowered into the centre of a vertical-bore, 11.7 T MR magnet (Magnex Scientific, Oxon, United Kingdom) with a 20 mm ^1^H birdcage coil (Rapid Biomedical, Würzburg, Germany) and a Bruker console (Bruker Medical, Ettlingen, Germany) running Paravision 2.1.1. Perfusion was then switched to a constant flow of 15-20 ml/min, based on each heart's coronary flow at 85 mmHg.

First-pass perfusion imaging was performed to cover the entire LV with repeated acquisitions using a T_1 _weighted fast gradient echo sequence that acquired one image every 128 ms (TE/TR, 0.8/2 ms; 60° pulse; field of view 20 × 20 mm; slice thickness 1 mm; matrix size 64 × 64, zero filled to 256 × 256). It was determined that rapid image acquisition was preferential to greater spatial resolution as this allowed more images to be acquired during the first pass of contrast agent, gave greater signal to noise and reduced motion artifacts. A bolus of 25 μl of 0.5 mmol/ml Gd-DTPA-BMA (Gadodiamine, Omniscan) was rapidly infused after approximately 10 of the 128 images were acquired. First-pass imaging was repeated during multiple bolus infusions to cover the entire LV. Control hearts were then removed from the magnet, the LAD was permanently occluded using a 5-0 silk suture (successful in 8 hearts) and first-pass imaging was repeated during multiple bolus infusions to cover the entire LV. Saturated gradient echo images were also acquired to determine whether LGE could be detected in the infarcted, perfused heart.

### Coronary occlusion model of myocardial infarction

Rats (n = 34) were anesthetized with 2.5% isoflurane in O_2_, intubated, and a thoracotomy was performed. The pericardium was removed and the heart was infarcted by ligation of the left anterior descending coronary artery (LAD), approximately 2 mm from the origin, using a 5-0 proline suture [26].

### *In vivo *CMR

Rats were anesthetized with 2.5% isoflurane in O_2 _and positioned supine in a purpose-built cradle. ECG electrodes were inserted into the forepaws, a respiration loop was taped across the chest and heart and respiration signals were monitored using a custom-built physiological motion gating device [27]. The cradle was lowered into a vertical-bore, 11.7 T MR system with a 52-mm birdcage coil (Rapid Biomedical, Würzburg, Germany) and a Bruker console running Paravision 2.1.1.

First-pass CMR was performed on a single short axis slice, which encompassed the papillary muscles, using a fast gradient echo sequence that acquired one image per heart beat (TE/TR, 0.8/2 ms; ~60° pulse; field of view 40 × 40 mm; slice thickness 1.5 mm; matrix size 64 × 64, zero filled to 256 × 256). The flip angle was adjusted to null signal in the myocardium before bolus infusion. A bolus of 0.5 mg/kg Gd in 200-270 μl saline was infused via the tail vein in 2 seconds during image acquisition. This volume, derived from *ex vivo *data, was ten times greater than that used in *ex vivo *experiments as *in vivo *cardiac output (75-100 ml/min) was 5 times greater than ex vivo perfusion rate (15 - 20 ml/min) and infusion time was 2 seconds *in vivo *and less than 1 second *ex vivo*

Cardiac cine-MRI was performed 10 to 25 min after Gd infusion as described [28]. A stack of contiguous 1.5 mm thick true short-axis ECG and respiration-gated cine-FLASH images (TE/TR 1.43/4.6 ms; 25° pulse; field of view 51.2 × 51.2 mm; matrix size 256 × 256; voxel size 200 × 200 × 1500 μm; 25 to 35 frames per cardiac cycle (mean heart rate 360 ± 20 bpm), were acquired to cover the entire left ventricle. Long-axis two-chamber and four-chamber images were also acquired. The relatively high flip angle (25°) of the cine-MRI acquisition allowed LGE-CMR to be assessed from the same stack of cine images, similar to the method recently described and validated by Protti *et al *[29]. The entire *in vivo *imaging protocol was performed in approximately 60 minutes.

### MRI data analysis

Image analysis was performed by an operator blinded to the surgical procedure performed on the rats using ImageJ (NIH Image, Bethesda, MD). Left ventricular volumes and ejection fractions were calculated from the stack of cine images as described [18]. To determine the area of akinetic scar tissue, the average endocardial and epicardial length of akinetic tissue in cine images were measured in each slice, summed across all slices and multiplied by slice thickness (1.5 mm) to give the absolute scar size as described by Nahrendorf et al [17] Perfusion was assessed by analyzing the change in signal intensity during Gd infusion in regions of interest (ROIs) selected in the infarcted anterior myocardium, adjacent to the infarct in the peri-infarcted lateral region and in the remote tissue of the inferior wall of the left ventricle. The relative rate of perfusion delay was measured as the time from inflow of contrast agent to the LV cavity, to 90% of the maximum signal intensity in the ROI. From *ex vivo *perfusion measurements, it was possible to measure the rate of contrast inflow and efflux from the gradient of the signal intensity/time curve. The area of reduced perfusion on the stack of *ex vivo *images was identified as myocardium with signal intensity greater than two standard deviations below that of remote myocardium.

*In vivo *LGE-MR images were thresholded to two standard deviations above the mean signal intensity from remote tissue. The volume and perimeter (endocardial length of the enhanced region) of the hyperenhanced region was traced manually and expressed as a percentage of total LV volume or perimeter.

### Data and statistical analysis

Results are shown as means ± standard errors. Differences were considered significant at *P *< 0.05 using unpaired Student t-tests or ANOVAs for repeated measures. Dependence between two measures was assessed using Pearson's Correlations.

## Results

### First-pass cardiac perfusion imaging in isolated, perfused rat hearts

**Figure 1:**
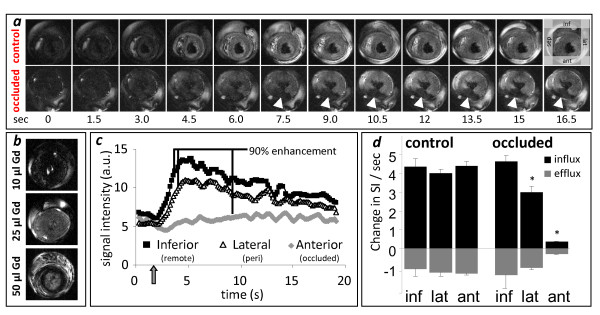
**First-pass imaging of the isolated, perfused rat heart**. **(*a*) **12 of the 128 images acquired prior to, during and after bolus Gd infusion from control and *ex vivo *occluded perfused hearts: **(*b*) **Images showing effect of different Gd bolus volumes; **(*c*) **Signal intensity/time curves from inferior (inf), lateral (lat) and anterior (ant) regions of a heart in which the coronary artery was occluded on the rig. Arrow indicates time of bolus infusion: **(*d*) **Graph of rates of change in signal intensity in different regions of control (n = 12) and *ex vivo *occluded (n = 8) hearts. Positive values calculated from contrast influx, negative values calculated from contrast efflux. *p < 0.05 compared with lateral remote tissue.

Repeated imaging of bolus injections of Gd were performed to determine the contrast agent infusion volume and duration that resulted in maximal contrast within perfused control rat hearts. At a buffer flow rate of 15 ml/min, a rapid (approximately 1 second) injection of 25 μl of 0.5 mmol/ml Gd, directly proximal to the aorta, led to uniform enhancement throughout the myocardium (Figure 1a, b), with time to 90% maximum enhancement similar for inferior, lateral and anterior regions. The rapid clearance of Gd from the tissue allowed bolus injections to be repeated every 90 seconds. Larger bolus infusions (> 50 μl) resulted in non uniform signal enhancement, and smaller infusion volumes (10 μl) did not enhance at all (Figure 1b). It was also possible to measure the gradient of the signal intensity/time curves at the time of Gd inflow. Again, the rate of signal intensity increase was similar for inferior, lateral and anterior regions (Figure 1d). The rate of clearance of the Gd bolus from the myocardium was calculated as the gradient of the signal intensity/time curve from 10 to 15 seconds after bolus infusion. No differences in Gd efflux between regions were observed (Figure 1d).

Perfused hearts were then removed from the magnet and the LAD occluded (successful occlusion in 8 of 12 hearts). Areas of low perfusion were identified below the occluded LAD as non-enhanced regions on MR images acquired during the Gd bolus infusion (Figure 1a). Perfusion delay (PD), calculated as the delay between time to 90% enhancement in an ROI compared with the remote inferior wall (Figure 1c), was significantly longer in the infarcted anterior wall (3.47 ± 0.65 s, p < 0.001) and the peri-infarcted lateral wall (0.81 ± 0.49 s, p < 0.05). The gradient of signal intensity increase was significantly lower in the infarcted (0.40 ± 0.02 a.u./s, p < 0.05) and peri infarcted (3.00 ± 0.20 a.u./s, p < 0.05) regions compared with remote regions (4.65 ± 0.30 a.u./s, Figure 1d). The rate of Gd clearance was also significantly lower in anterior myocardial regions affected by LAD occlusion (infarct, -0.28 ± 0.04, peri-infarct -1.00 ± 0.20, remote -1.40 ± 0.68 a.u./s, Figure 1d). Saturated gradient echo images, acquired up to 50 min after coronary occlusion found no evidence of LGE in the perfused infarcted hearts.

### *In vivo *first-pass perfusion imaging of infarcted rat hearts

#### Cardiac morphology and function

Cine-MRI measurements of cardiac morphology and function were made serially on rats prior to and at 2, 8 and 42 days after infarction (Table 1). Left ventricular mass, end diastolic volume (EDV), end systolic volume (ESV) and body weight were significantly increased at 8 and 42 days after infarction. Ejection fraction (EF) was reduced by 2 days, whereas stroke volume and cardiac output were depressed at 2 days, but returned to normal levels by 8 days. The percentage of akinetic myocardium and heart rates did not change from 2 to 42 days.

**Table 1:** *In vivo *measurements of rat heart morphology and function before and at 2, 8 and 42 days after infarction (n = 27)

	Pre	2 days	8 days	42 days
Left ventricular mass (mg)	492 ± 35	543 ± 18*	571 ± 15*	586 ± 16*^#^
End diastolic volume (μl)	265 ± 12	282 ± 11	395 ± 15*^#^	428 ± 24*^#+^
End systolic volume (μl)	55 ± 8	108 ± 5*	154 ± 13*^#^	185 ± 19*^#+^
Stroke volume (μl)	210 ± 9	174 ± 8*	241 ± 7^#^	243 ± 11^#^
Ejection fraction (%)	80 ± 2	62 ± 1*	62 ± 2*	58 ± 2*^#+^
Akinetic myocardium (mm^2^)	-	16 ± 4*	23 ± 5*^#^	28 ± 6*^#+^
Akinetic myocardium (%)	-	9 ± 2*	10 ± 2*	11 ± 2*
Heart rate (bpm)	404 ± 11	361 ± 9	344 ± 27	346 ± 26
Cardiac output (ml/min)	85 ± 5	61 ± 3*	83 ± 3^#^	80 ± 3^#^
Weight (g)	234 ± 11	214 ± 3	230 ± 5	245 ± 19

* p < 0.05 compared with pre-infarction. ^#^p < 0.05 compared with 2 days. ^+ ^p < 0.05 compared with 8 days,

#### First-pass cardiac perfusion imaging 2 and 8 days post infarction

**Figure 2:**
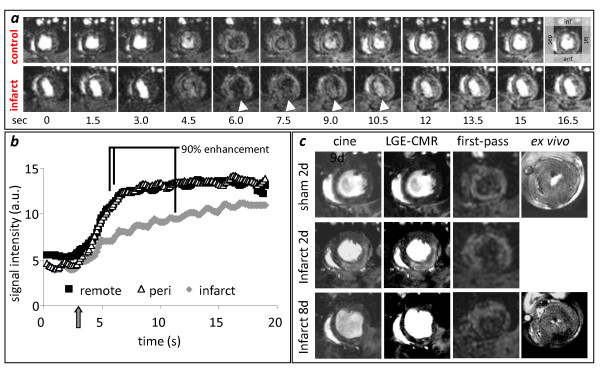
***In vivo *first-pass imaging**. **(*a*) **12 of the 128 images acquired prior to, during and after bolus Gd infusion from control and *in vivo *infarcted rat hearts: **(*b*) **signal intensity/time curves from infarcted, peri-infarcted and remote regions of an infarcted heart. Arrow indicates time of bolus infusion: **(*c*) ***in vivo *cine-CMR, LGE-CMR and first-pass images from a sham operated rat and an infarcted rat at 2 and 8 days post surgery. First-pass images from the same isolated, perfused hearts are presented on the far right.

Infusion of Gd initially reduced signal in the LV cavity owing to T1 and T2 shortening of blood from the high concentration of contrast agent (Figure 2a). This made it impossible to measure an arterial input function. However, signal in the myocardium became enhanced shortly after cavity hypoenhancement, as contrast agent perfused through the coronary circulation. Slight cyclical elevations in myocardial signal intensity occurred due to respiratory motion. Even though the image acquisition time continued through much of the cardiac cycle, cardiac motion did not prevent measurement of contrast inflow. Imaging of first-pass perfusion in rat hearts identified areas of perfusion deficit in the region affected by the LAD coronary artery occlusion (Figure 2a, b). The average perfusion delay in infarct and peri-infarct compared with remote tissue was 4.0 ± 0.7 and 1.7 ± 0.4 s at 2 days post infarction and 3.3 ± 0.5 and 0.5 ± 0.3 s at 8 days post infarction.

#### Late Gadolinium enhancement imaging 2 and 8 days post infarction

LGE-CMR was performed 10-25 min after first-pass imaging. Hyperenhancement was present in 18 ± 2% of the LV volume and 19 ± 2% of LV endocardial perimeter at 2 days post infarction, and 11 ± 1% of the LV volume and 16 ± 2% of LV endocardial perimeter at 8 days post infarction (Figure 2c).

**Figure 3:**
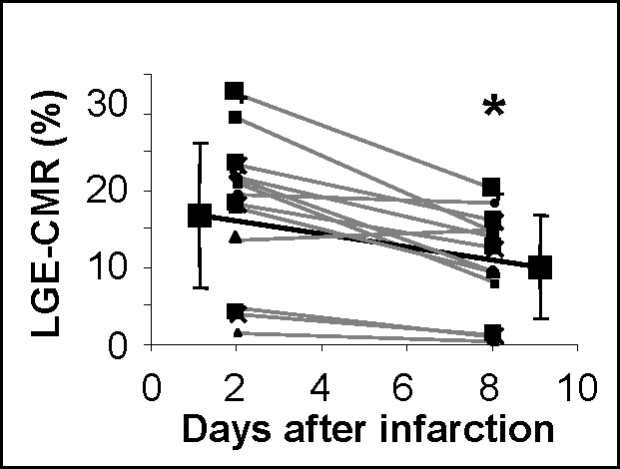
**Repeated measures of LGE-CMR volume made in the same 14 infarcted rat hearts that had successful tail vein cannulation at 2 and 8 days after infarction**. * *p *< 0.05 compared with 2 day data.

For the sub-group of infarcted rats (n = 14) in which tail vein canulation was successful at both time points, first-pass CMR and LGE-CMR were performed at both 2 and 8 days post infarction. Perfusion delay (measured using first-pass CMR) and LGE-CMR endocardial scar perimeter were unchanged from 2 to 8 days (Table 2). However, there was a significant reduction in LGE-CMR scar size (measured as % LV volume) between 2 and 8 days (19 ± 3 and 10 ± 2% respectively, *p *< 0.01; Figure 3 and Table 2) and in absolute scar size (100 ± 18 μl at 2 days vs. 56 ± 11 μl at 8 days, *p *< 0.01).

**Table 2:** *In vivo *measurements of cardiac perfusion and viability in infarcted rat hearts (n = 14) at 2 and 8 days after infarction

	2 days	8 days
Perfusion delay (s)	3.57 ± 0.71	3.51 ± 0.58
LGE-CMR % perimeter	18 ± 3	15 ± 3
LGE-CMR % volume	19 ± 3	10 ± 2^#^
LGE-CMR volume (μl)	100 ± 18	56 ± 11^#^

^#^p < 0.01 compared with 2 days.

#### Correlations between MRI measurements at 2 and 8 days and ejection fractions and end systolic volumes at 42 days

**Figure 4:**
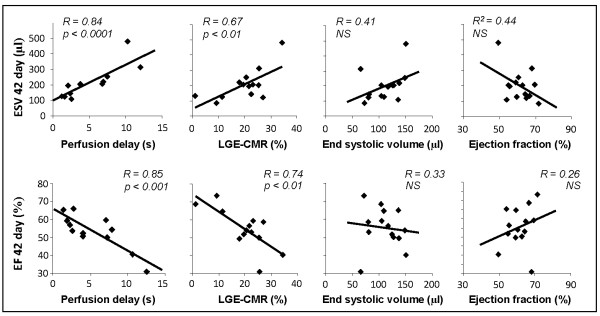
**Correlations between 2 day measurements of perfusion delay, LGE-CMR, ESV or EF, and resulting ESV or EF measured in the same animals at 42 days post infarction (n - 14 animals that had successful tail vein cannulation at 2 days and were imaged at 42 days)**.

Perfusion delays measured at 2 days post infarction correlated with left ventricular end systolic volumes (*R = 0.84, P < 0.0001*) and ejection fractions (*R = 0.85, P *< 0.001) measured at 42 days (Figure 4), suggesting that rats with acute perfusion deficit went on to develop greater chronic cardiac impairment. As anticipated, infarct size measured using LGE-CMR at 2 days post infarction correlated with left ventricular end systolic volumes (*R = 0.67, P < 0.01*) and ejection fractions (*R = 0.74, P < 0.01*) measured at 42 days (Figure 4). However, correlations between infarct size and EF or ESV were lower than those found using first-pass CMR. At 2 days, perfusion delay gave a better prediction of outcome than cine MRI measurements of morphology and function, with no correlation between 2 day end systolic volumes and ejection fractions and the end systolic volumes and ejection fractions measured at 42 days. Perfusion delay measured at 8 days post infarction also correlated with ESV (*R = 0.76, P *< 0.05) and EF (*R = 0.63, P *< 0.05) at 42 days. Again, these correlations were stronger than those between LGE-CMR, ESV and EF at 8 days and the resulting ejection fractions and end systolic volumes measured at 42 days (data not shown).

#### First-pass cardiac perfusion imaging in isolated, perfused infarcted rat hearts

Nine days after infarction, 7 rat hearts were excised, perfused in Langendorff mode and imaged. Infusion of 25 μl Gd into the perfusate proximal to the aorta gave uniform signal enhancement throughout the remote myocardium, whereas infarcted regions showed only limited enhancement (Figure 2c). Signal intensity/time curves plotted from images acquired at the same location *in vivo *and *ex vivo *showed similar characteristics and resulted in similar measurements of perfusion delay (*in vivo *= 2.9 ± 2.1s, *ex vivo *= 2.8 ± 0.8s, correlation; *R^2 ^*= 0.7. *p *< 0.05). The gradient of signal intensity increase was significantly lower in the infarcted and peri infarcted regions compared with remote tissue (infarct, 0.38 ± 0.03, peri 2.36 ± 0.42, remote 4.31 ± 0.91, p < 0.01), while the rate of Gd clearance was significantly lower in infarcted regions compared with remote (infarct, 0.05 ± 0.04, peri 0.25 ± 0.08, remote 0.44 ± 0.05). These experiments also indicated that the volume of reduced perfusion measured *ex vivo *matched the volume of LGE measured *in vivo *(Additional file 1). Interestingly, saturated gradient echo images performed 10 to 50 minutes after Gd infusion found no evidence of LGE in the perfused infarcted hearts. This probably occurred because Gd was infused as a single 25 μl bolus and heart perfusate was not circulated, preventing accumulation of Gd in the infarcted tissue

## Discussion

Clinical studies have demonstrated that the extent of myocardial perfusion deficit after infarction is a strong predictor of functional outcome, with patients who present with large microvascular obstructions (MVO) having poor prognosis [10]. MR imaging of the first-pass of a bolus infusion of Gd is now a well established technique for accurate identification and localisation of MVOs [5,11-13,30]. Despite its importance, first-pass MR imaging has only very recently been used in small animal models of myocardial infarction [24,25]. Here we developed a CMR method which acquired one image every heart cycle and was sensitive to the inflow of Gd into the myocardium, thus providing a rapid technique for assessing tissue perfusion.

Several methods have been used to assess myocardial perfusion in small animal models. Arterial spin labelling and T_1 _mapping can provide quantitative measurement of blood flow post infarction in rats and mice [20-23]. However, the accuracy of these techniques is decreased by rapid and variable heart rates, artefacts produced by respiratory motion during acquisition and a single slice acquisition takes tens of minutes. PET imaging of radiotracers can be used to measure myocardial perfusion, but low spatial resolution, attenuation artefacts and the need for a local cyclotron to produce tracers, such as ^82^Rb or H_2_^15^O, limit the application of this technology, especially when applied to small animals [5,11-13,30,31].

We initially developed and optimised the first-pass method using isolated perfused rat hearts. This preparation permitted repeated contrast infusions in the same heart so that Gd bolus volume, concentration and infusion time could be optimised in a controlled environment. Repeated infusions indicated good reproducibility and uniform enhancement in anterior, lateral and inferior myocardial regions. To determine the sensitivity of the first-pass technique to regional perfusion deficits the LAD coronary artery was occluded and first-pass imaging was repeated. Signal intensity was measured in regions of interest placed in infarcted, peri-infarcted and remote tissue of all 128 images acquired during bolus infusion. Two methods were used to quantify regional perfusion changes. Firstly, perfusion delay was calculated as the time delay, in seconds, between 90% max enhancement in infarcted regions compared with remote regions. Secondly, in ex vivo perfused hearts it was possible to assess contrast influx rate from the linear portion of the positive gradient of the signal intensity/time curve and contrast efflux rate from the signal intensity/time curve 10-15 seconds after infusion. As both of these analysis methods relate changes in infarcted regions with those of remote regions of the same infusion any effects of slight differences in bolus transit time and volume can be reduced, providing more reproducible data.

The first-pass imaging method had sufficient T_1 _weighting and temporal and spatial resolution to permit in vivo measurement of contrast inflow into the myocardium. The requirement for a rapid imaging sequence that could acquire an image every heart beat favoured a fast gradient echo acquisition rather than the more traditional inversion recovery sequence. The fast gradient echo acquisition resulted in signal saturation within the myocardium prior to contrast arrival, but did not null signal in the blood pool due to the rapid influx. Contrast transit through the ventricular cavities reduced signal intensity owing to the susceptibility effects of high Gd concentration at 11.7 T. Importantly the Gd bolus resulted in signal enhancement within the perfused myocardial tissue. As with the isolated perfused heart experiments, signal intensity was measured in regions of interest placed in infarcted, peri-infarcted and remote tissue of all 128 images acquired during bolus infusion and perfusion delay was calculated as the time delay in seconds between 90% max enhancement in infarcted regions compared with remote regions. Perfusion delay in the infarcted regions was significantly longer than in remote tissue. Owing to the need for acquisition of high temporally resolved images, the *in vivo *spatial resolution of the first-pass technique is limited. Although regions of interest could be selected within the infarct territory, it was not possible to accurately assess the area of the perfusion deficit. This is augmented in the thinned scar tissue of the chronically infarcted heart and can make delineation of the endocardial border problematic. A further limitation of the method is that it cannot be performed at multiple slice positions during a single Gd bolus, while repeated bolus imaging is complicated by LGE in infarcted regions.

The severity of perfusion delay and extent of delayed enhancement measured at 2 and 8 days strongly correlated with cardiac function measured in the same rats 42 days after infarction. These correlations were stronger than those between ejection fraction and end systolic volume measured at 2 or 8 days and ejection fraction or end systolic volume at 42 days. Acutely after infarction, myocardial stunning, edema, and elevated catecholamine levels can strongly influence EF and ESV, masking the extent of systolic dysfunction. A combination of these factors, coupled with the variability of infarct size typically induced by experimental myocardial infarction, makes acute measurement of EF and ESV a poor early predictor of outcome and suggests that the common practice of dividing infarcted animals into experimental groups based on EF measured made principle by echocardiography in the first week post infarction can lead to wide variability in extent of functional impairment between groups. The first-pass and LGE-CMR techniques presented here gave better predictors of outcome cine-MRI, provided a more accurate assessment of myocardial viability acutely after infarction and may offer a useful baseline measure of infarct severity prior to administration of the therapeutic agent.

E*x vivo *first-pass imaging was performed on isolated, perfused infarcted rat hearts that had undergone *in vivo *imaging at 2 and 8 days post infarction. *In vivo *and *ex vivo *measurements of perfusion delay correlated, as did *in vivo *LGE-CMR volume and *ex vivo *low perfusion volume and Gd clearance time. This supports the hypothesis that LGE-CMR is due to reduced washout and hence greater retention of Gd in infarcted regions [32].

The volume of LGE significantly decreased from 2 days to 8 days post infarction, and was unquantifiable at 42 days. Similar results were reported in canine models [33] and in two recent clinical studies which found a decrease in LGE from 12 to 8% of the left ventricle between 1 and 8 days after infarction [34] and from 27 to 22% between 24 hours to 6 months [35]. This decrease is probably due to hyperenhancement of oedematous, inflamed, viable tissue within the infarct border zone at 2 days post infarction, and thinning of the scar, infarct resorption and hypertrophy of remote tissue at 8 days [33,34]. However, it draws attention to possible limitations of using LGE-CMR in small animal models of chronic infarction where transmurality and tissue remodelling can make infarct assessment difficult. Few studies have used LGE-CMR chronically after infarction, and those that have did not study animals with large transmural infarcts [36], The first-pass CMR measurements of perfusion delays measured in this study did not change from 2 to 8 days post infarction, but we predict that the spatial resolution of the technique may be inadequate for measuring perfusion in established transmural scar tissue.

The resolution of the first-pass method may be improved through parallel imaging using multi channel phase array coils and accelerated image acquisitions. Schneider et al recently demonstrated the feasibility of using parallel imaging to achieve threefold acceleration of cine image acquisition in mice [37]. By using similar strategies, it may be possible to perform multi slice first-pass imaging with a spatial resolution that permits accurate delineation of underperfused areas of myocardium, or to assess changes in perfusion in peri-infarct areas in response to dobutamine stress or after experimental therapy with angiogenic cytokines or stem cells. A novel therapeutic strategy for replacing myocardium lost during infarction is grafting of engineered heart tissue [38,39]. It is currently difficult to assess *in vivo *whether the graft is incorporated into the host's vasculature. First-pass CMR may be able to detect contrast agent inflow into the engineered tissue and determine successful integration.

## Conclusions

In summary, we have presented a novel, simple and useful MR method for identification of under-perfused tissue in the infarcted rat heart and demonstrate the high prognostic value of the technique compared with other non-invasive measures of cardiac function and viability. This method is a useful tool for assessing the severity of myocardial ischemia acutely after infarction and could provide important data on treatment strategies designed to revascularise and regenerate the infarcted heart.

## Abbreviations

LGE-CMR: late gadolinium enhancement cardiac magnetic resonance; EF: ejection fraction; EDV: end diastolic volume; ESV: end systolic volume; FLASH: fast low angle shot; Gd-DTPA: gadolinium diethylenetriaminepentaacetate; LAD: left anterior descending coronary artery; LV: left ventricle; MVO: microvascular obstruction; PD: pefusion delay.

## Competing interests

The authors declare that they have no competing interests.

## Authors' contributions

DJS conceived and designed the study, performed all MRI and analysis, and drafted the manuscript; CAC performed all surgical procedures and assisted with MRI; SJM and MAC assisted with perfused heart experiments, DJT assisted with MRI; KC assisted with study design, manuscript preparation and funding. All authors read and approved the final manuscript

## Supplementary Material

Additional file 1 **(*a*) Plot showing similarity in percent volume of LGE-MRI in slices from base to apex measured *in vivo *8 days post infarction, and percent volume of myocardium with low perfusion measured from base to apex measured using first-pass MRI of isolated, perfused rat hearts 9 days post infarction: (*b*) Correlation between LGE-MRI volumes measured *in vivo *and low perfusion volumes measured using first-pass MRI in the same slice in isolated, perfused hearts (R = 0.80. *P *< 0.01)**. Click here for file
