# Supplementary figures and images for: First-pass perfusion CMR two days after infarction predicts severity of functional impairment six weeks later in the rat heart
Source: J Cardiovasc Magn Reson. 2011 Aug 3;13(1):38. doi: 10.1186/1532-429X-13-38 (PMC3162911; doi:10.1186/1532-429X-13-38)

# Supplementary Figure S1

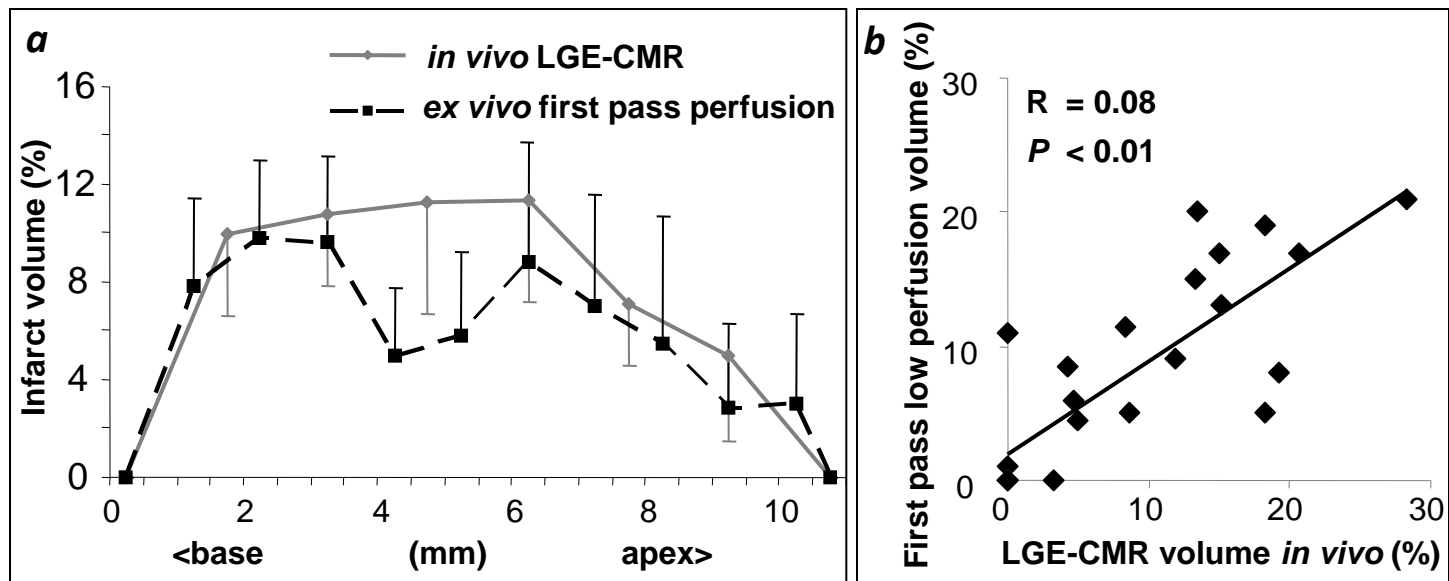

Supplement: Additional file 1 — (a) Plot showing similarity in percent volume of LGE-MRI in slices from base to apex measured in vivo 8 days post infarction, and percent volume of myocardium with low perfusion measured from base to apex measured using first-pass MRI of isolated, perfused rat hearts 9 days post infarction: (b) Correlation between LGE-MRI volumes measured in vivo and low perfusion volumes measured using first-pass MRI in the same slice in isolated, perfused hearts (R = 0.80. P < 0.01). [file 1532-429X-13-38-S1.PDF]
